# Supplementary material for: Assessing the response to genomic selection by simulation
Source: Theor Appl Genet. 2022 Jul 14;135(8):2891–905. doi: 10.1007/s00122-022-04157-1 (PMC9325815; doi:10.1007/s00122-022-04157-1)
Supplement: Supplementary file 6 — Supplementary file6 (PDF 224 kb) [file 122_2022_4157_MOESM6_ESM.pdf]

Response to Selection based on Genomic Prediction Based on a Simulation

This is the code to simulate response to selection based on genomic prediction and to generate the plot. The dataset used here is the pollen pool GCA1 data.

Load the R packages

```
library(parallel)
library(foreach)
library(doParallel)
library(tictoc)
library(plyr)
library(doSNOW)
library(progress)
library(reshape2)

rm(list = ls())
options(scipen = 999, digits = 5)
```

Reload Gamma Matrix

```
Gamma.mat <- readRDS("Gamma_Mat_GCA1_MY.RDS")
```

Entries

```
##### Number of Entries #####
row.gamma <- nrow(Gamma.mat)
n.geno <- row.gamma/2

# Take n (proportion selection)
n.1 <- round(n.geno*0.01,0)
n.5 <- round(n.geno*0.05,0)
n.10 <- round(n.geno*0.10,0)
n.15 <- round(n.geno*0.15,0)
n.20 <- round(n.geno*0.20,0)
n.30 <- round(n.geno*0.30,0)
n.50 <- round(n.geno*0.50,0)
```

Parallelisation setup

```
##### Parallelisation setup #####
n.cores <- detectCores() - 1
```

```r
my.cluster <- parallel::makeCluster(n.cores, type = "PSOCK")
print(my.cluster)
```

```
## Socketcluster mit 7 Knoten auf System 'localhost'
```

```r
registerDoSNOW(my.cluster)

iterations <- 100000

##### Function for separate the results in the simulation #####
comb <- function(x, ...) {
  lapply(seq_along(x),
         function(i) c(x[[i]], lapply(list(...), function(y) y[[i]])))
}

##### Progress Time #####
pb <- progress_bar$new(format = ":percent | Elapsed time: :elapsed eta: :eta",
                       total = iterations, clear = FALSE)

progress <- function(n){
  pb$tick()
}

opts <- list(progress = progress)
```

Start Simulation

```r
##### Start Simulation #####
tic()
simu.list <- foreach(i = 1:iterations,
                     .combine = comb, .multicombine = TRUE,
                     .options.snow = opts,
                     .init = list(list(), list(),list(),list(),list(),list(),list(),list())) %dopar% {

    z <- matrix(rnorm(n = row.gamma),nrow = row.gamma)

    w <- Gamma.mat %*% z # W matrix

    sim.ghat <- w[((row.gamma/2) + 1):row.gamma,] # Take the Ghat
    sim.gtrue <- w[1:(row.gamma/2),] # Take the true genotype values


    # Make a data frame for Ghat and true genotype values
    v <- data.frame(sim.ghat = sim.ghat, sim.gtrue = sim.gtrue,
                    rank.ghat = rank(-sim.ghat), rank.gtrue = rank(-sim.gtrue), nsim = i)

    # Subset the data from the simu^^lation for each n
    sel.1  <- v[v$rank.ghat <= n.1, ]
    sel.5  <- v[v$rank.ghat <= n.5, ]
    sel.10 <- v[v$rank.ghat <= n.10, ]
    sel.15 <- v[v$rank.ghat <= n.15, ]
    sel.20 <- v[v$rank.ghat <= n.20, ]
    sel.30 <- v[v$rank.ghat <= n.30, ]
    sel.50 <- v[v$rank.ghat <= n.50, ]
```

```r
sel.1$n  <- n.1
sel.5$n  <- n.5
sel.10$n <- n.10
sel.15$n <- n.15
sel.20$n <- n.20
sel.30$n <- n.30
sel.50$n <- n.50

# Count the best entries
# Sel 1%
top1.sel1  <- ifelse(length(which(sel.1$rank.gtrue %in% 1 == TRUE)) == 1, 1, 0)
top3.sel1  <- ifelse(length(which(sel.1$rank.gtrue %in% c(1:3) == TRUE)) == 3, 1, 0)
top5.sel1  <- ifelse(length(which(sel.1$rank.gtrue %in% c(1:5) == TRUE)) == 5, 1, 0)
top10.sel1 <- ifelse(length(which(sel.1$rank.gtrue %in% c(1:10) == TRUE)) == 10, 1, 0)
top15.sel1 <- ifelse(length(which(sel.1$rank.gtrue %in% c(1:15) == TRUE)) == 15, 1, 0)

# Sel 5%
top1.sel5  <- ifelse(length(which(sel.5$rank.gtrue %in% 1 == TRUE)) == 1, 1, 0)
top3.sel5  <- ifelse(length(which(sel.5$rank.gtrue %in% c(1:3) == TRUE)) == 3, 1, 0)
top5.sel5  <- ifelse(length(which(sel.5$rank.gtrue %in% c(1:5) == TRUE)) == 5, 1, 0)
top10.sel5 <- ifelse(length(which(sel.5$rank.gtrue %in% c(1:10) == TRUE)) == 10, 1, 0)
top15.sel5 <- ifelse(length(which(sel.5$rank.gtrue %in% c(1:15) == TRUE)) == 15, 1, 0)

top1.sel10  <- ifelse(length(which(sel.10$rank.gtrue %in% 1 == TRUE)) == 1, 1, 0)
top3.sel10  <- ifelse(length(which(sel.10$rank.gtrue %in% c(1:3) == TRUE)) == 3, 1, 0)
top5.sel10  <- ifelse(length(which(sel.10$rank.gtrue %in% c(1:5) == TRUE)) == 5, 1, 0)
top10.sel10 <- ifelse(length(which(sel.10$rank.gtrue %in% c(1:10) == TRUE)) == 10, 1, 0)
top15.sel10 <- ifelse(length(which(sel.10$rank.gtrue %in% c(1:15) == TRUE)) == 15, 1, 0)

# Sel 15%
top1.sel15  <- ifelse(length(which(sel.15$rank.gtrue %in% 1 == TRUE)) == 1, 1, 0)
top3.sel15  <- ifelse(length(which(sel.15$rank.gtrue %in% c(1:3) == TRUE)) == 3, 1, 0)
top5.sel15  <- ifelse(length(which(sel.15$rank.gtrue %in% c(1:5) == TRUE)) == 5, 1, 0)
top10.sel15 <- ifelse(length(which(sel.15$rank.gtrue %in% c(1:10) == TRUE)) == 10, 1, 0)
top15.sel15 <- ifelse(length(which(sel.15$rank.gtrue %in% c(1:15) == TRUE)) == 15, 1, 0)

# Sel 20%
top1.sel20  <- ifelse(length(which(sel.20$rank.gtrue %in% 1 == TRUE)) == 1, 1, 0)
top3.sel20  <- ifelse(length(which(sel.20$rank.gtrue %in% c(1:3) == TRUE)) == 3, 1, 0)
top5.sel20  <- ifelse(length(which(sel.20$rank.gtrue %in% c(1:5) == TRUE)) == 5, 1, 0)
top10.sel20 <- ifelse(length(which(sel.20$rank.gtrue %in% c(1:10) == TRUE)) == 10, 1, 0)
top15.sel20 <- ifelse(length(which(sel.20$rank.gtrue %in% c(1:15) == TRUE)) == 15, 1, 0)

# Sel 30%
top1.sel30  <- ifelse(length(which(sel.30$rank.gtrue %in% 1 == TRUE)) == 1, 1, 0)
top3.sel30  <- ifelse(length(which(sel.30$rank.gtrue %in% c(1:3) == TRUE)) == 3, 1, 0)
top5.sel30  <- ifelse(length(which(sel.30$rank.gtrue %in% c(1:5) == TRUE)) == 5, 1, 0)
top10.sel30 <- ifelse(length(which(sel.30$rank.gtrue %in% c(1:10) == TRUE)) == 10, 1, 0)
top15.sel30 <- ifelse(length(which(sel.30$rank.gtrue %in% c(1:15) == TRUE)) == 15, 1, 0)

# Sel 50%
top1.sel50  <- ifelse(length(which(sel.50$rank.gtrue %in% 1 == TRUE)) == 1, 1, 0)
top3.sel50  <- ifelse(length(which(sel.50$rank.gtrue %in% c(1:3) == TRUE)) == 3, 1, 0)
top5.sel50  <- ifelse(length(which(sel.50$rank.gtrue %in% c(1:5) == TRUE)) == 5, 1, 0)
top10.sel50 <- ifelse(length(which(sel.50$rank.gtrue %in% c(1:10) == TRUE)) == 10, 1, 0)
top15.sel50 <- ifelse(length(which(sel.50$rank.gtrue %in% c(1:15) == TRUE)) == 15, 1, 0)
```

```r
    # sel 1% results
    best.sel1  <- data.frame(nsim = i, sel = n.1, n.top1 = top1.sel1,
                             n.top3 = top3.sel1, n.top5 = top5.sel1,
                             n.top10 = top10.sel1, n.top15 = top15.sel1)

    # sel 5% results
    best.sel5  <- data.frame(nsim = i, sel = n.5, n.top1 = top1.sel5,
                             n.top3 = top3.sel5, n.top5 = top5.sel5,
                             n.top10 = top10.sel5, n.top15 = top15.sel5)

    # sel 10% results
    best.sel10  <- data.frame(nsim = i, sel = n.10, n.top1 = top1.sel10,
                              n.top3 = top3.sel10, n.top5 = top5.sel10,
                              n.top10 = top10.sel10, n.top15 = top15.sel10)

    # sel 15% results
    best.sel15  <- data.frame(nsim = i, sel = n.15, n.top1 = top1.sel15,
                              n.top3 = top3.sel15, n.top5 = top5.sel15,
                              n.top10 = top10.sel15, n.top15 = top15.sel15)

    # sel 20% results
    best.sel20  <- data.frame(nsim = i, sel = n.20, n.top1 = top1.sel20,
                              n.top3 = top3.sel20, n.top5 = top5.sel20,
                              n.top10 = top10.sel20, n.top15 = top15.sel20)

    # sel 30% results
    best.sel30  <- data.frame(nsim = i, sel = n.30, n.top1 = top1.sel30,
                              n.top3 = top3.sel30, n.top5 = top5.sel30,
                              n.top10 = top10.sel30, n.top15 = top15.sel30)

    # sel 50% results
    best.sel50  <- data.frame(nsim = i, sel = n.50, n.top1 = top1.sel50,
                              n.top3 = top3.sel50, n.top5 = top5.sel50,
                              n.top10 = top10.sel50, n.top15 = top15.sel50)

    list(v, best.sel1, best.sel5, best.sel10,
         best.sel15, best.sel20, best.sel30, best.sel50)
  }
toc()
```

```
## 1028.91 sec elapsed
```

Data wrangling the results

```r
##### Extract imulated data #####
tic()
df <- simu.list[[1]]
df <- dplyr::bind_rows(df, .id = "nsim")
toc()
```

```
## 9.22 sec elapsed
```

```r
#####  Correlations from the simulated data #####
cor(df$sim.ghat, df$sim.gtrue) # Correlation GBLUPs with the true values
```

```
## [1] 0.65308
```

```r
cor(df$rank.gtrue, df$rank.ghat) # Correlation GBLUPs ranking with the true values ranking
```

```
## [1] 0.63068
```

```r
##### Extract the data for each selection proprotion #####
sel1  <- simu.list[[2]]
sel5  <- simu.list[[3]]
sel10 <- simu.list[[4]]
sel15 <- simu.list[[5]]
sel20 <- simu.list[[6]]
sel30 <- simu.list[[7]]
sel50 <- simu.list[[8]]

sel1  <- dplyr::bind_rows(sel1 , .id = "nsim")
sel5  <- dplyr::bind_rows(sel5 , .id = "nsim")
sel10 <- dplyr::bind_rows(sel10, .id = "nsim")
sel15 <- dplyr::bind_rows(sel15, .id = "nsim")
sel20 <- dplyr::bind_rows(sel20, .id = "nsim")
sel30 <- dplyr::bind_rows(sel30, .id = "nsim")
sel50 <- dplyr::bind_rows(sel50, .id = "nsim")

##### Stack into one data frame #####
sel_dat <- rbind(sel1, sel5, sel10, sel15,
                 sel20, sel30, sel50)

##### Stop cluster for parallel #####
stopCluster(my.cluster)
```

Compute Probability

```r
prob.best <- data.frame(dummy = 1)
tic()
for (i in 1:5) {

  f <- as.formula((paste(names(sel_dat[i + 2]), "~ sel")))
  pro <- aggregate(f, sel_dat, FUN = "mean")

  if (i > 1) {
    pro <- pro[-1]
  }
  prob.best <- cbind(prob.best, pro)

  if (i == 1) {
    prob.best <- prob.best[-1]
  }
}
toc()
```

```
## 3.62 sec elapsed
```

Data preparation for plot

```r
## Transpose the dataset by n
final.dat <- melt(prob.best, id = "sel")
names(final.dat)[names(final.dat) == 'sel'] <- 'n'
```

```r
final.dat$n <- as.numeric(final.dat$n)

## Change the second column name
names(final.dat)[2] <- "m"

## Make a column for genotype numbers
final.dat$n.geno <- n.geno

## Make a column for the x axis of the plot (selection proportion)
final.dat$n.pct <- round(final.dat$n/final.dat$n.geno,3)*100

final.dat$m <- as.character(final.dat$m)

## Rename the rows in column m
final.dat$m <- ifelse(final.dat$m == "n.top1" , "Best 1",
                      ifelse(final.dat$m == "n.top3" , "Best 3",
                             ifelse(final.dat$m == "n.top5" , "Best 5",
                                    ifelse(final.dat$m == "n.top10" , "Best 10", "Best 15"))))

final.dat$m <- as.factor(final.dat$m)

## Arrange the level for making the plot
final.dat$m <- factor(final.dat$m,
                      levels = c("Best 1", "Best 3", "Best 5", "Best 10", "Best 15"))

# Make column for identifier of the pool and the cycles
# It is not necessary if only one plot to be made
final.dat$Pool <- "Pollen pool"
final.dat$Cycle <- "GCA1-MY"

knitr::kable(final.dat)
```

n	m	value	n.geno	n.pct	Pool	Cycle
19	Best 1	0.41931	1910	1	Pollen pool	GCA1-MY
96	Best 1	0.73234	1910	5	Pollen pool	GCA1-MY
191	Best 1	0.85175	1910	10	Pollen pool	GCA1-MY
286	Best 1	0.90682	1910	15	Pollen pool	GCA1-MY
382	Best 1	0.93885	1910	20	Pollen pool	GCA1-MY
573	Best 1	0.97218	1910	30	Pollen pool	GCA1-MY
955	Best 1	0.99416	1910	50	Pollen pool	GCA1-MY
19	Best 3	0.07521	1910	1	Pollen pool	GCA1-MY
96	Best 3	0.36842	1910	5	Pollen pool	GCA1-MY
191	Best 3	0.58305	1910	10	Pollen pool	GCA1-MY
286	Best 3	0.71302	1910	15	Pollen pool	GCA1-MY
382	Best 3	0.80055	1910	20	Pollen pool	GCA1-MY
573	Best 3	0.90099	1910	30	Pollen pool	GCA1-MY
955	Best 3	0.97684	1910	50	Pollen pool	GCA1-MY
19	Best 5	0.01567	1910	1	Pollen pool	GCA1-MY
96	Best 5	0.18281	1910	5	Pollen pool	GCA1-MY
191	Best 5	0.38828	1910	10	Pollen pool	GCA1-MY
286	Best 5	0.54505	1910	15	Pollen pool	GCA1-MY
382	Best 5	0.66508	1910	20	Pollen pool	GCA1-MY
573	Best 5	0.82059	1910	30	Pollen pool	GCA1-MY
955	Best 5	0.95345	1910	50	Pollen pool	GCA1-MY
19	Best 10	0.00041	1910	1	Pollen pool	GCA1-MY

n	m	value	n.geno	n.pct	Pool	Cycle
96	Best 10	0.03264	1910	5	Pollen pool	GCA1-MY
191	Best 10	0.13550	1910	10	Pollen pool	GCA1-MY
286	Best 10	0.26758	1910	15	Pollen pool	GCA1-MY
382	Best 10	0.40258	1910	20	Pollen pool	GCA1-MY
573	Best 10	0.62662	1910	30	Pollen pool	GCA1-MY
955	Best 10	0.88539	1910	50	Pollen pool	GCA1-MY
19	Best 15	0.00001	1910	1	Pollen pool	GCA1-MY
96	Best 15	0.00605	1910	5	Pollen pool	GCA1-MY
191	Best 15	0.04705	1910	10	Pollen pool	GCA1-MY
286	Best 15	0.12764	1910	15	Pollen pool	GCA1-MY
382	Best 15	0.23548	1910	20	Pollen pool	GCA1-MY
573	Best 15	0.46491	1910	30	Pollen pool	GCA1-MY
955	Best 15	0.80838	1910	50	Pollen pool	GCA1-MY

Make the plot

```
library(ggplot2)
p <- ggplot(final.dat, aes(x = n.pct, y = value)) +
  geom_line(aes(x = n.pct, y = value, color = m, group = m)) +
  geom_point(aes(color = m, group = m)) +
  geom_label(aes(x = 0.5, y = 1.06, label = paste("N =", n.geno)),
             size    = 3.5,
             data    = final.dat,
             hjust   = 0,
             vjust   = 0.5
  ) +
  labs(caption = "Number of simulations = S = 100K") +
  scale_y_continuous(name = "Probability of selection", breaks = seq(0, 1, 0.10)) +
  scale_x_continuous(name = "Number of selected entries (n)",
                     breaks = c(1, 5, 10, 15, 20, 30, 50),
                     labels = scales::percent_format(scale = 1, accuracy = 1)) +
  scale_colour_brewer(palette = "Set1", name = "Number of truly best entries (m)") +
  theme_bw() +
  theme(
    panel.grid.minor.x = element_blank(),
    panel.grid.major.x = element_blank(),
    panel.background = element_blank(),
    plot.caption = element_text(size = 11),
    axis.title.y = element_text(face = "bold", size = 12),
    strip.text.x = element_text(size = 12, face = "bold"),
    strip.text.y = element_text(size = 12, face = "bold"),
    axis.title.x = element_text(face = "bold", size = 12,lineheight = 1),
    axis.text = element_text(angle = 0, hjust = 0.5, face = "bold", size = 10),
    legend.text = element_text(size = 11, face = "bold"),
    title = element_text(face = "bold", size = 11),legend.position = "bottom")

p
```

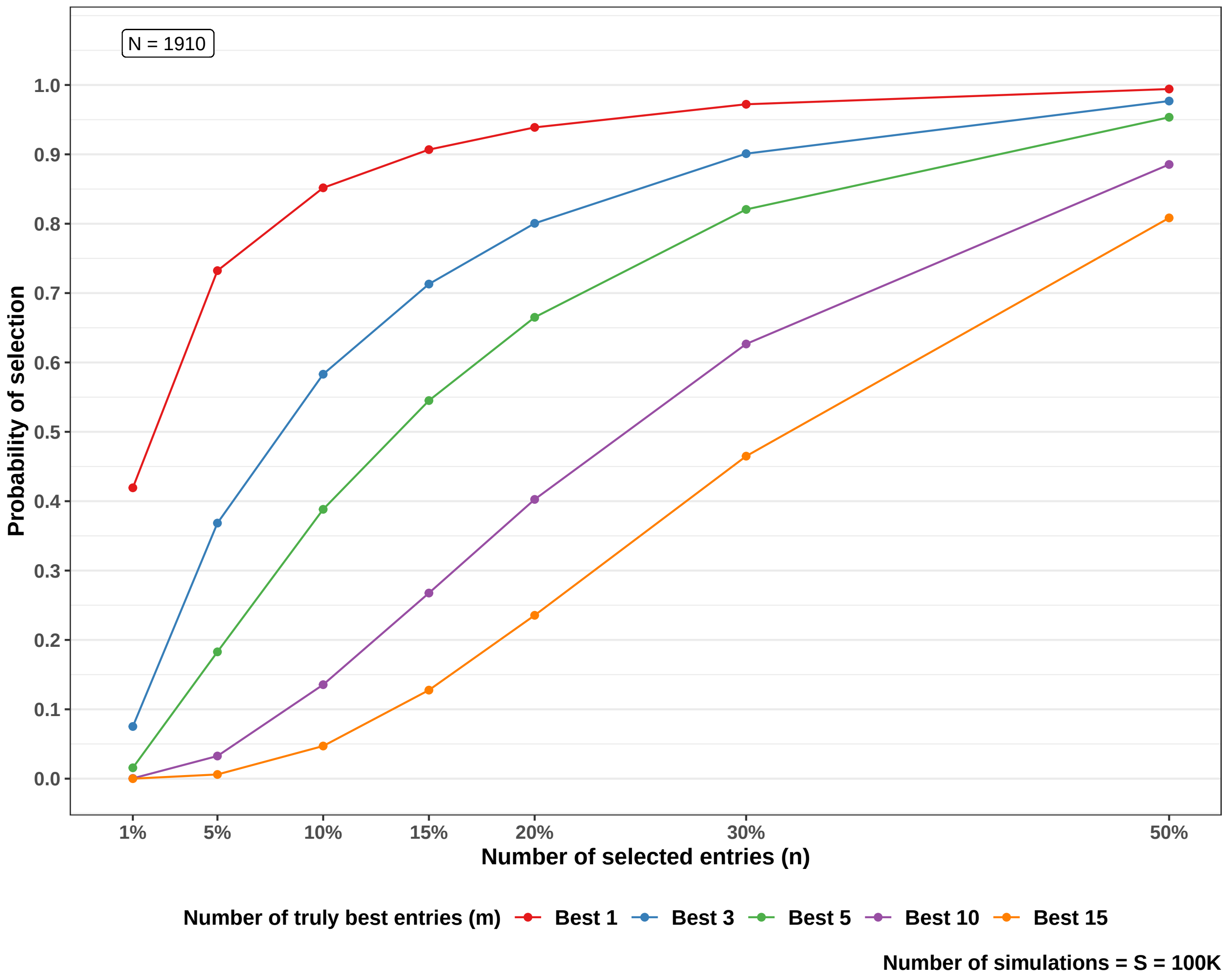
N = 1910
Probability of selection
0.0
0.1
0.2
0.3
0.4
0.5
0.6
0.7
0.8
0.9
1.0
1%
5%
10%
15%
20%
30%
50%
Number of selected entries (n)
Number of truly best entries (m)
Best 1
Best 3
Best 5
Best 10
Best 15
Number of simulations = S = 100K
