# Supplementary material for: Assessing the response to genomic selection by simulation
Source: Theor Appl Genet. 2022 Jul 14;135(8):2891–905. doi: 10.1007/s00122-022-04157-1 (PMC9325815; doi:10.1007/s00122-022-04157-1)
Supplement: Supplementary file 8 — Supplementary file8 (HTML 1750 kb) [file 122_2022_4157_MOESM8_ESM.html]

Response to Selection based on Genomic Prediction Based on a Simulation (Seed Pool GCA\_MY)


- Load the R
  packages
- Reload Gamma
  Matrix
- Entries
- Parallelisation
  setup
- Start Simulation
- Data wrangling the
  results
- Compute
  Probability
- Data preparation
  for plot
- Make the plot

# Response to Selection based on Genomic Prediction Based on a Simulation (Seed Pool GCA\_MY)

This is the code to simulate response to selection based on genomic
prediction and to generate the plot. The dataset used here is the seed
pool GCA1 data.

# Load the R packages

```
library(parallel)
library(foreach)
library(doParallel)
library(tictoc)
library(plyr)
library(doSNOW)
library(progress)
library(reshape2)

rm(list = ls())
options(scipen = 999, digits = 5)
```

# Reload Gamma Matrix

```
Gamma.mat <- readRDS("Gamma_Mat_GCA1_MY.RDS")
```

# Entries

```
##### Number of Entries ##### 
row.gamma <- nrow(Gamma.mat)
row.gamma
```

```
## [1] 2256
```

```
n.geno <- row.gamma/2

# Take n (proportion selection)
n.1 <- round(n.geno*0.01,0)
n.5 <- round(n.geno*0.05,0)
n.10 <- round(n.geno*0.10,0)
n.15 <- round(n.geno*0.15,0)
n.20 <- round(n.geno*0.20,0)
n.30 <- round(n.geno*0.30,0)
n.50 <- round(n.geno*0.50,0)
```

# Parallelisation setup

```
##### Parallelisation setup #####
n.cores <- detectCores() - 1

my.cluster <- parallel::makeCluster(n.cores, type = "PSOCK")
print(my.cluster)
```

```
## socket cluster with 7 nodes on host 'localhost'
```

```
registerDoSNOW(my.cluster)

iterations <- 100000

##### Function for separate the results in the simulation #####
comb <- function(x, ...) {
  lapply(seq_along(x),
         function(i) c(x[[i]], lapply(list(...), function(y) y[[i]])))
}

##### Progress Time ##### 
pb <- progress_bar$new(format = ":percent | Elapsed time: :elapsed eta: :eta",
                       total = iterations, clear = FALSE)

progress <- function(n){
  pb$tick()
} 

opts <- list(progress = progress)
```

# Start Simulation

```
##### Start Simulation ##### 
tic()
simu.list <- foreach(i = 1:iterations, 
                     .combine = comb, .multicombine = TRUE, 
                     .options.snow = opts,
                     .init = list(list(), list(), list(), list(), 
                                  list(), list(), list(), list())) %dopar% {
    
    z <- matrix(rnorm(n = row.gamma),nrow = row.gamma)
    
    w <- Gamma.mat %*% z # W matrix
    
    sim.ghat <- w[((row.gamma/2) + 1):row.gamma,] # Take the Ghat
    sim.gtrue <- w[1:(row.gamma/2),] # Take the true genotype values
    
    
    # Make a data frame for Ghat and true genotype values
    v <- data.frame(sim.ghat = sim.ghat, sim.gtrue = sim.gtrue, 
                    rank.ghat = rank(-sim.ghat), rank.gtrue = rank(-sim.gtrue), nsim = i)
    
    # Subset the data from the simu^^lation for each n
    sel.1  <- v[v$rank.ghat <= n.1, ]
    sel.5  <- v[v$rank.ghat <= n.5, ]
    sel.10 <- v[v$rank.ghat <= n.10, ]
    sel.15 <- v[v$rank.ghat <= n.15, ]
    sel.20 <- v[v$rank.ghat <= n.20, ]
    sel.30 <- v[v$rank.ghat <= n.30, ]
    sel.50 <- v[v$rank.ghat <= n.50, ]
    
    sel.1$n  <- n.1
    sel.5$n  <- n.5
    sel.10$n <- n.10
    sel.15$n <- n.15
    sel.20$n <- n.20
    sel.30$n <- n.30
    sel.50$n <- n.50
    
    # Count the best entries
    # Sel 1%
    top1.sel1  <- ifelse(length(which(sel.1$rank.gtrue %in% 1 == TRUE)) == 1, 1, 0)
    top3.sel1  <- ifelse(length(which(sel.1$rank.gtrue %in% c(1:3) == TRUE)) == 3, 1, 0)
    top5.sel1  <- ifelse(length(which(sel.1$rank.gtrue %in% c(1:5) == TRUE)) == 5, 1, 0)
    top10.sel1 <- ifelse(length(which(sel.1$rank.gtrue %in% c(1:10) == TRUE)) == 10, 1, 0)
    top15.sel1 <- ifelse(length(which(sel.1$rank.gtrue %in% c(1:15) == TRUE)) == 15, 1, 0)
    
    # Sel 5%
    top1.sel5  <- ifelse(length(which(sel.5$rank.gtrue %in% 1 == TRUE)) == 1, 1, 0)
    top3.sel5  <- ifelse(length(which(sel.5$rank.gtrue %in% c(1:3) == TRUE)) == 3, 1, 0)
    top5.sel5  <- ifelse(length(which(sel.5$rank.gtrue %in% c(1:5) == TRUE)) == 5, 1, 0)
    top10.sel5 <- ifelse(length(which(sel.5$rank.gtrue %in% c(1:10) == TRUE)) == 10, 1, 0)
    top15.sel5 <- ifelse(length(which(sel.5$rank.gtrue %in% c(1:15) == TRUE)) == 15, 1, 0)
    
    top1.sel10  <- ifelse(length(which(sel.10$rank.gtrue %in% 1 == TRUE)) == 1, 1, 0)
    top3.sel10  <- ifelse(length(which(sel.10$rank.gtrue %in% c(1:3) == TRUE)) == 3, 1, 0)
    top5.sel10  <- ifelse(length(which(sel.10$rank.gtrue %in% c(1:5) == TRUE)) == 5, 1, 0)
    top10.sel10 <- ifelse(length(which(sel.10$rank.gtrue %in% c(1:10) == TRUE)) == 10, 1, 0)
    top15.sel10 <- ifelse(length(which(sel.10$rank.gtrue %in% c(1:15) == TRUE)) == 15, 1, 0)
    
    # Sel 15%
    top1.sel15  <- ifelse(length(which(sel.15$rank.gtrue %in% 1 == TRUE)) == 1, 1, 0)
    top3.sel15  <- ifelse(length(which(sel.15$rank.gtrue %in% c(1:3) == TRUE)) == 3, 1, 0)
    top5.sel15  <- ifelse(length(which(sel.15$rank.gtrue %in% c(1:5) == TRUE)) == 5, 1, 0)
    top10.sel15 <- ifelse(length(which(sel.15$rank.gtrue %in% c(1:10) == TRUE)) == 10, 1, 0)
    top15.sel15 <- ifelse(length(which(sel.15$rank.gtrue %in% c(1:15) == TRUE)) == 15, 1, 0)
    
    # Sel 20%
    top1.sel20  <- ifelse(length(which(sel.20$rank.gtrue %in% 1 == TRUE)) == 1, 1, 0)
    top3.sel20  <- ifelse(length(which(sel.20$rank.gtrue %in% c(1:3) == TRUE)) == 3, 1, 0)
    top5.sel20  <- ifelse(length(which(sel.20$rank.gtrue %in% c(1:5) == TRUE)) == 5, 1, 0)
    top10.sel20 <- ifelse(length(which(sel.20$rank.gtrue %in% c(1:10) == TRUE)) == 10, 1, 0)
    top15.sel20 <- ifelse(length(which(sel.20$rank.gtrue %in% c(1:15) == TRUE)) == 15, 1, 0)
    
    # Sel 30%
    top1.sel30  <- ifelse(length(which(sel.30$rank.gtrue %in% 1 == TRUE)) == 1, 1, 0)
    top3.sel30  <- ifelse(length(which(sel.30$rank.gtrue %in% c(1:3) == TRUE)) == 3, 1, 0)
    top5.sel30  <- ifelse(length(which(sel.30$rank.gtrue %in% c(1:5) == TRUE)) == 5, 1, 0)
    top10.sel30 <- ifelse(length(which(sel.30$rank.gtrue %in% c(1:10) == TRUE)) == 10, 1, 0)
    top15.sel30 <- ifelse(length(which(sel.30$rank.gtrue %in% c(1:15) == TRUE)) == 15, 1, 0)
    
    # Sel 50%
    top1.sel50  <- ifelse(length(which(sel.50$rank.gtrue %in% 1 == TRUE)) == 1, 1, 0)
    top3.sel50  <- ifelse(length(which(sel.50$rank.gtrue %in% c(1:3) == TRUE)) == 3, 1, 0)
    top5.sel50  <- ifelse(length(which(sel.50$rank.gtrue %in% c(1:5) == TRUE)) == 5, 1, 0)
    top10.sel50 <- ifelse(length(which(sel.50$rank.gtrue %in% c(1:10) == TRUE)) == 10, 1, 0)
    top15.sel50 <- ifelse(length(which(sel.50$rank.gtrue %in% c(1:15) == TRUE)) == 15, 1, 0)
    
    # sel 1% results
    best.sel1  <- data.frame(nsim = i, sel = n.1, n.top1 = top1.sel1, 
                             n.top3 = top3.sel1, n.top5 = top5.sel1,
                             n.top10 = top10.sel1, n.top15 = top15.sel1)
    
    # sel 5% results
    best.sel5  <- data.frame(nsim = i, sel = n.5, n.top1 = top1.sel5, 
                             n.top3 = top3.sel5, n.top5 = top5.sel5,
                             n.top10 = top10.sel5, n.top15 = top15.sel5)
    
    # sel 10% results
    best.sel10  <- data.frame(nsim = i, sel = n.10, n.top1 = top1.sel10, 
                              n.top3 = top3.sel10, n.top5 = top5.sel10,
                              n.top10 = top10.sel10, n.top15 = top15.sel10)
    
    # sel 15% results
    best.sel15  <- data.frame(nsim = i, sel = n.15, n.top1 = top1.sel15, 
                              n.top3 = top3.sel15, n.top5 = top5.sel15,
                              n.top10 = top10.sel15, n.top15 = top15.sel15)
    
    # sel 20% results
    best.sel20  <- data.frame(nsim = i, sel = n.20, n.top1 = top1.sel20, 
                              n.top3 = top3.sel20, n.top5 = top5.sel20,
                              n.top10 = top10.sel20, n.top15 = top15.sel20)
    
    # sel 30% results
    best.sel30  <- data.frame(nsim = i, sel = n.30, n.top1 = top1.sel30, 
                              n.top3 = top3.sel30, n.top5 = top5.sel30,
                              n.top10 = top10.sel30, n.top15 = top15.sel30)
    
    # sel 50% results
    best.sel50  <- data.frame(nsim = i, sel = n.50, n.top1 = top1.sel50, 
                              n.top3 = top3.sel50, n.top5 = top5.sel50,
                              n.top10 = top10.sel50, n.top15 = top15.sel50)
    
    list(v, best.sel1, best.sel5, best.sel10,
         best.sel15, best.sel20, best.sel30, best.sel50)
  }
toc()
```

```
## 423.42 sec elapsed
```

# Data wrangling the results

```
##### Extract imulated data #####
tic()
df <- simu.list[[1]]
df <- dplyr::bind_rows(df, .id = "nsim")
toc()
```

```
## 6.9 sec elapsed
```

```
#####  Correlations from the simulated data ##### 
cor(df$sim.ghat, df$sim.gtrue) # Correlation GBLUPs with the true values
```

```
## [1] 0.6882
```

```
cor(df$rank.gtrue, df$rank.ghat) # Correlation GBLUPs ranking with the true values ranking
```

```
## [1] 0.66447
```

```
##### Extract the data for each selection proprotion ##### 
sel1  <- simu.list[[2]]
sel5  <- simu.list[[3]]
sel10 <- simu.list[[4]]
sel15 <- simu.list[[5]]
sel20 <- simu.list[[6]]
sel30 <- simu.list[[7]]
sel50 <- simu.list[[8]]

sel1  <- dplyr::bind_rows(sel1 , .id = "nsim")
sel5  <- dplyr::bind_rows(sel5 , .id = "nsim")
sel10 <- dplyr::bind_rows(sel10, .id = "nsim")
sel15 <- dplyr::bind_rows(sel15, .id = "nsim")
sel20 <- dplyr::bind_rows(sel20, .id = "nsim")
sel30 <- dplyr::bind_rows(sel30, .id = "nsim")
sel50 <- dplyr::bind_rows(sel50, .id = "nsim")

##### Stack into one data frame ##### 
sel_dat <- rbind(sel1, sel5, sel10, sel15,
                 sel20, sel30, sel50)

##### Stop cluster for parallel #####
stopCluster(my.cluster)
```

# Compute Probability

```
prob.best <- data.frame(dummy = 1)
tic()  
for (i in 1:5) {
  
  f <- as.formula((paste(names(sel_dat[i + 2]), "~ sel")))
  pro <- aggregate(f, sel_dat, FUN = "mean")
  
  if (i > 1) { 
    pro <- pro[-1]
  }
  prob.best <- cbind(prob.best, pro)
  
  if (i == 1) { 
    prob.best <- prob.best[-1]
  }
}
toc()
```

```
## 3.46 sec elapsed
```

# Data preparation for plot

```
## Transpose the dataset by n
final.dat <- melt(prob.best, id = "sel")
names(final.dat)[names(final.dat) == 'sel'] <- 'n'
final.dat$n <- as.numeric(final.dat$n)  

## Change the second column name
names(final.dat)[2] <- "m"

## Make a column for genotype numbers
final.dat$n.geno <- n.geno

## Make a column for the x axis of the plot (selection proportion)
final.dat$n.pct <- round(final.dat$n/final.dat$n.geno,3)*100

final.dat$m <- as.character(final.dat$m)

## Rename the rows in column m
final.dat$m <- ifelse(final.dat$m == "n.top1" , "Best 1",
                      ifelse(final.dat$m == "n.top3" , "Best 3",
                             ifelse(final.dat$m == "n.top5" , "Best 5",
                                    ifelse(final.dat$m == "n.top10" , "Best 10", "Best 15"))))

final.dat$m <- as.factor(final.dat$m)

## Arrange the level for making the plot
final.dat$m <- factor(final.dat$m,
                      levels = c("Best 1", "Best 3", "Best 5", "Best 10", "Best 15"))

# Make column for identifier of the pool and the cycles
# It is not necessary if only one plot to be made
final.dat$Pool <- "Pollen pool"
final.dat$Cycle <- "GCA1-MY"

knitr::kable(final.dat)
```

| n | m | value | n.geno | n.pct | Pool | Cycle |
| --- | --- | --- | --- | --- | --- | --- |
| 11 | Best 1 | 0.43787 | 1128 | 1 | Pollen pool | GCA1-MY |
| 56 | Best 1 | 0.75135 | 1128 | 5 | Pollen pool | GCA1-MY |
| 113 | Best 1 | 0.86644 | 1128 | 10 | Pollen pool | GCA1-MY |
| 169 | Best 1 | 0.91821 | 1128 | 15 | Pollen pool | GCA1-MY |
| 226 | Best 1 | 0.94704 | 1128 | 20 | Pollen pool | GCA1-MY |
| 338 | Best 1 | 0.97579 | 1128 | 30 | Pollen pool | GCA1-MY |
| 564 | Best 1 | 0.99447 | 1128 | 50 | Pollen pool | GCA1-MY |
| 11 | Best 3 | 0.07262 | 1128 | 1 | Pollen pool | GCA1-MY |
| 56 | Best 3 | 0.38547 | 1128 | 5 | Pollen pool | GCA1-MY |
| 113 | Best 3 | 0.60588 | 1128 | 10 | Pollen pool | GCA1-MY |
| 169 | Best 3 | 0.73396 | 1128 | 15 | Pollen pool | GCA1-MY |
| 226 | Best 3 | 0.81751 | 1128 | 20 | Pollen pool | GCA1-MY |
| 338 | Best 3 | 0.90856 | 1128 | 30 | Pollen pool | GCA1-MY |
| 564 | Best 3 | 0.97754 | 1128 | 50 | Pollen pool | GCA1-MY |
| 11 | Best 5 | 0.01083 | 1128 | 1 | Pollen pool | GCA1-MY |
| 56 | Best 5 | 0.18817 | 1128 | 5 | Pollen pool | GCA1-MY |
| 113 | Best 5 | 0.40424 | 1128 | 10 | Pollen pool | GCA1-MY |
| 169 | Best 5 | 0.56404 | 1128 | 15 | Pollen pool | GCA1-MY |
| 226 | Best 5 | 0.68334 | 1128 | 20 | Pollen pool | GCA1-MY |
| 338 | Best 5 | 0.83061 | 1128 | 30 | Pollen pool | GCA1-MY |
| 564 | Best 5 | 0.95581 | 1128 | 50 | Pollen pool | GCA1-MY |
| 11 | Best 10 | 0.00004 | 1128 | 1 | Pollen pool | GCA1-MY |
| 56 | Best 10 | 0.02856 | 1128 | 5 | Pollen pool | GCA1-MY |
| 113 | Best 10 | 0.13398 | 1128 | 10 | Pollen pool | GCA1-MY |
| 169 | Best 10 | 0.26882 | 1128 | 15 | Pollen pool | GCA1-MY |
| 226 | Best 10 | 0.40727 | 1128 | 20 | Pollen pool | GCA1-MY |
| 338 | Best 10 | 0.63178 | 1128 | 30 | Pollen pool | GCA1-MY |
| 564 | Best 10 | 0.88435 | 1128 | 50 | Pollen pool | GCA1-MY |
| 11 | Best 15 | 0.00000 | 1128 | 1 | Pollen pool | GCA1-MY |
| 56 | Best 15 | 0.00423 | 1128 | 5 | Pollen pool | GCA1-MY |
| 113 | Best 15 | 0.04276 | 1128 | 10 | Pollen pool | GCA1-MY |
| 169 | Best 15 | 0.12040 | 1128 | 15 | Pollen pool | GCA1-MY |
| 226 | Best 15 | 0.22916 | 1128 | 20 | Pollen pool | GCA1-MY |
| 338 | Best 15 | 0.45664 | 1128 | 30 | Pollen pool | GCA1-MY |
| 564 | Best 15 | 0.80127 | 1128 | 50 | Pollen pool | GCA1-MY |

# Make the plot

```
library(ggplot2)
p <- ggplot(final.dat, aes(x = n.pct, y = value)) + 
  geom_line(aes(x = n.pct, y = value, color = m, group = m)) +
  geom_point(aes(color = m, group = m)) +
  geom_label(aes(x = 0.5, y = 1.06, label = paste("N =", n.geno)),
             size    = 3.5,
             data    = final.dat,
             hjust   = 0,
             vjust   = 0.5
  ) +
  labs(caption = "Number of simulations = S = 100K") +
  scale_y_continuous(name = "Probability of selection", breaks = seq(0, 1, 0.10)) +
  scale_x_continuous(name = "Number of selected entries (n)", 
                     breaks = c(1, 5, 10, 15, 20, 30, 50),
                     labels = scales::percent_format(scale = 1, accuracy = 1)) +
  scale_colour_brewer(palette = "Set1", name = "Number of truly best entries (m)") +
  theme_bw() +
  theme(
    panel.grid.minor.x = element_blank(),
    panel.grid.major.x = element_blank(),
    panel.background = element_blank(),
    plot.caption = element_text(size = 11),
    axis.title.y = element_text(face = "bold", size = 12),
    strip.text.x = element_text(size = 12, face = "bold"),
    strip.text.y = element_text(size = 12, face = "bold"),
    axis.title.x = element_text(face = "bold", size = 12,lineheight = 1),
    axis.text = element_text(angle = 0, hjust = 0.5, face = "bold", size = 10),
    legend.text = element_text(size = 11, face = "bold"),
    title = element_text(face = "bold", size = 11),legend.position = "bottom")

p
```
