# Supplementary material for: Assessing the response to genomic selection by simulation
Source: Theor Appl Genet. 2022 Jul 14;135(8):2891–905. doi: 10.1007/s00122-022-04157-1 (PMC9325815; doi:10.1007/s00122-022-04157-1)
Supplement: Supplementary file 12 — Supplementary file11 (PDF 144 kb) [file 122_2022_4157_MOESM12_ESM.pdf]

Supplementary Tables

Table S.1. Variance estimates and standard errors for year (σ_y^2), GBV (σ_g^2), GBV×year interaction effects (σ_{gy}^2) in CYC and MY based on the main year fixed effect model.

Pool	Variance	MY		CYC	
		Estimate	SE*	Estimate	SE*
Seed pool					
	σ_g^2	1.961	0.203	1.271	0.242
	σ_{gy}^2	1.405	0.151	4.720	0.280
Pollen pool					
	σ_g^2	3.339	0.280	4.267	0.289
	σ_{gy}^2	3.366	0.221	3.120	0.187

*SE, standard error

Table S.2. The $\hat{\rho}$ of the CYC and the MY analyses based on the main year fixed effect model.

Pool	MY		CYC	
	$\hat{\rho}$	SE*	$\hat{\rho}$	SE*
Seed pool	0.582	0.044	0.213	0.038
Pollen pool	0.498	0.032	0.578	0.026

*SE, standard error

Table S.3. Variance estimates and standard errors for year (σ_y^2), GBV (σ_g^2), GBV×year interaction effects (σ_{gy}^2) for each cycle of the GCA2 assessment based on the main year fixed effect model.

Pool	Variance	Estimate and standard error (SE)							
		Cycle 1		Cycle 2		Cycle 3		Cycle 4	
		Estimate	SE*	Estimate	SE*	Estimate	SE*	Estimate	SE*
Seed pool									
	σ_g^2	0.633	0.321	0.697	0.228	1.306	0.338	0.320	0.212
	σ_{gy}^2	0.486	0.251	0.086	0.125	0.000	-	0.245	0.187
Pollen pool									
	σ_g^2	4.267	0.754	0.707	0.206	1.707	0.388	0.396	0.165
	σ_{gy}^2	0.782	0.305	0.136	0.111	0.452	0.200	0.193	0.141

*SE, standard error
